# RAD51AP1 mediates RAD51 activity through nucleosome interaction

**DOI:** 10.1016/j.jbc.2021.100844

**Published:** 2021-05-28

**Authors:** Elena Pires, Neelam Sharma, Platon Selemenakis, Bo Wu, Yuxin Huang, Dauren S. Alimbetov, Weixing Zhao, Claudia Wiese

**Affiliations:** 1 Department of Environmental and Radiological Health Sciences, Colorado State University, Fort Collins, Colorado, USA; 2 Cell and Molecular Biology Graduate Program, Colorado State University, Fort Collins, Colorado, USA; 3 Department of Biochemistry and Structural Biology, University of Texas Health San Antonio, San Antonio, Texas, USA

**Keywords:** homologous recombination DNA repair, chromatin, mononucleosomes, synaptic complex assembly, RAD51AP1, RAD51, AA, amino acid, ANOVA, analysis of variance, ATP, adenosine triphosphate, BSA, bovine serum albumin, D-loop, displacement-loop, EMSA, electrophoretic mobility shift assay, HPLC, high-pressure liquid chromatography, HR, homologous recombination, Hop2, homologous pairing 2, MBP, maltose-binding protein, MMC, mitomycin C, MNase, micrococcal nuclease, Mnd1, meiotic nuclear divisions 1, NAP1, nucleosome-associated protein 1, NCP, nucleosome core particle, Ni-NTA, nickel nitrilotriacetic acid, PALB2, partner and localizer of BRCA2, PVDF, polyvinylidene difluoride, SEC, size-exclusion chromatography, UAF1, USP1-associated factor 1, RAD51AP1, RAD51-associated protein 1

## Abstract

RAD51-associated protein 1 (RAD51AP1) is a key protein in the homologous recombination (HR) DNA repair pathway. Loss of RAD51AP1 leads to defective HR, genome instability, and telomere erosion. RAD51AP1 physically interacts with the RAD51 recombinase and promotes RAD51-mediated capture of donor DNA, synaptic complex assembly, and displacement-loop formation when tested with nucleosome-free DNA substrates. In cells, however, DNA is packaged into chromatin, posing an additional barrier to the complexities of the HR reaction. In this study, we show that RAD51AP1 binds to nucleosome core particles (NCPs), the minimum basic unit of chromatin in which approximately two superhelical turns of 147 bp double-stranded DNA are wrapped around one histone octamer with no free DNA ends remaining. We identified a C-terminal region in RAD51AP1, including its previously mapped DNA-binding domain, as critical for mediating the association between RAD51AP1 and both the NCP and the histone octamer. Using *in vitro* surrogate assays of HR activity, we show that RAD51AP1 is capable of promoting duplex DNA capture and initiating joint-molecule formation with the NCP and chromatinized template DNA, respectively. Together, our results suggest that RAD51AP1 directly assists in the RAD51-mediated search for donor DNA in chromatin. We present a model, in which RAD51AP1 anchors the DNA template through affinity for its nucleosomes to the RAD51-ssDNA nucleoprotein filament.

Homologous recombination DNA repair (HR) is a template-dependent DNA damage repair pathway critical for genome stability and cancer avoidance. HR also is essential for the smooth progression of DNA replication (1). Central to the HR reaction is the RAD51-ssDNA nucleoprotein filament, also called the presynaptic filament, which captures the DNA donor, engages in synapsis, and generates a displacement-loop (D-loop) upon location of the homologous DNA target sequence.

RAD51AP1 is an intrinsically unfolded protein (2) and is likely to undergo induced folding upon binding to specific partners, which will then make it well-ordered (3). RAD51AP1 binds to the RAD51 recombinase and stimulates RAD51-mediated joint-molecule formation, as we and others have shown (4, 5, 6, 7, 8, 9, 10). In RAD51AP1, two distinct DNA-binding domains have been identified (4, 6). Both domains are required for the full activity of RAD51AP1 in protecting cells from DNA damaging agents and in joint-molecule formation assays with synthetic DNA substrates *in vitro* (6).

Previous efforts to elucidate the biochemical properties of RAD51AP1 relied on nucleosome-free DNA substrates (4, 5, 6, 7, 8, 9). In eukaryotic cells, however, DNA is packaged into chromatin with nucleosomes forming the minimum basic unit. How RAD51AP1 would promote RAD51 activity in the context of reconstituted nucleosome core particles (NCPs) and nucleosome-containing donor DNA was unclear.

In this study, we show that RAD51AP1 physically associates with the NCP. Binding to the NCP retains the RAD51AP1-RAD51 interaction. In the context of the NCP, RAD51AP1 stimulates the capture of the DNA template. RAD51AP1 also stimulates RAD51-mediated strand invasion into chromatinized DNA. Collectively, our results suggest that RAD51AP1 is an important accessory HR factor in the chromatin environment and functions as a bridging molecule between the RAD51-ssDNA nucleoprotein filament and the incoming homologous chromatin donor. We also show that a previously identified DNA-binding domain in RAD51AP1 is critical for establishing the contact with the NCP and strand invasion.

## Results

### RAD51AP1 interacts with the mononucleosome core particle (NCP)

**Figure 1:**
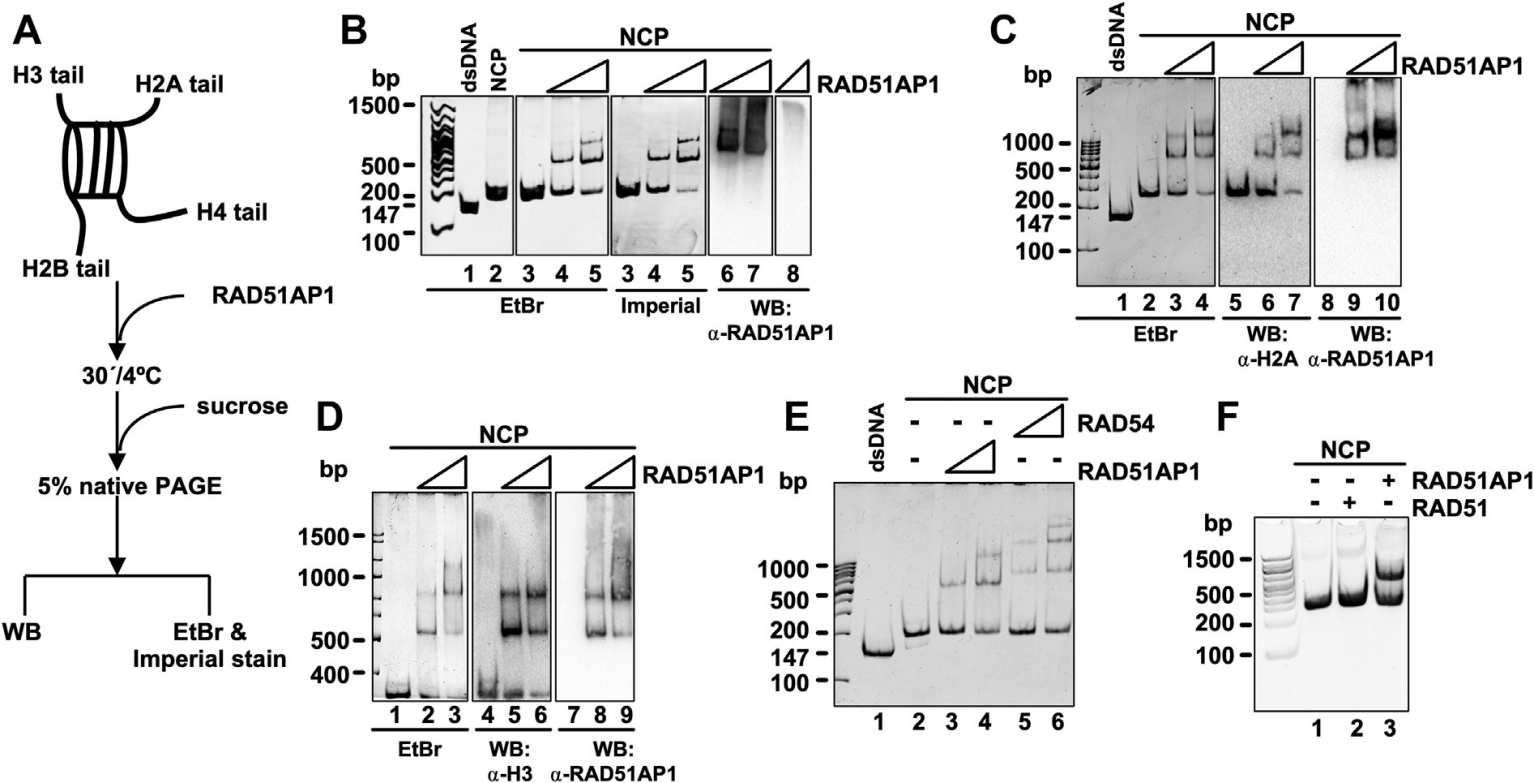
**RAD51AP1 interacts with the nucleosome core particle (NCP).***A*, schematic of the NCP and the EMSA protocol. *B*, EMSA of RAD51AP1-His_6_ (0.1 and 0.2 μM) with the NCP (0.2 μM) assessed by ethidium bromide (lanes 3–5) first and then by Imperial protein stain (lanes 3–5). The western blots show the presence of RAD51AP1 entering the gel only when bound to the NCP (lanes 6–7), not alone (lane 8). *C*, EMSA of His_6_/FLAG-tagged RAD51AP1 (0.4 and 0.8 μM) with the NCP (0.4 μM) visualized by ethidium bromide (lanes 1–4). A second gel was loaded in duplicate, transferred, and probed to histone H2A or RAD51AP1. *D*, EMSA of His_6_/FLAG-tagged RAD51AP1 (0.4 and 0.8 μM) with the NCP (0.4 μM) visualized by ethidium bromide (lanes 1–3). A second gel was loaded in duplicate, transferred, and probed to histone H3 or RAD51AP1. *E*, EMSA of His6/FLAG-tagged RAD51AP1 (0.1 and 0.2 μM) and of FLAG-tagged RAD54 (0.1 and 0.2 μM) with the NCP (0.2 μM) assessed by ethidium bromide (lanes 3–4 and 5–6, respectively). *F*, RAD51 does not bind to the NCP. EMSA of RAD51 and RAD51AP1 (0.4 and 0.2 μM, respectively) with the NCP (0.2 μM).

Intrigued by our earlier findings that showed affinity of purified human RAD51AP1 to chromatinized DNA in the immobilized template assay (11), we decided to test complex formation between RAD51AP1 and the NCP. The NCP is the minimum basic unit of chromatin in which approximately two superhelical turns of 147 bp double-stranded (ds)DNA are wrapped around one histone octamer with no free DNA ends remaining (12, 13). We reconstituted NCPs by salt gradient deposition using the 601 Widom 147 bp dsDNA fragment and human histone octamers, as previously described (12, 14), and used electrophoretic mobility shift assays (EMSAs) to assess the RAD51AP1-NCP association (for schematic of the assay see Fig. 1*A*). Following incubation of full-length human His_6_-tagged RAD51AP1 (Fig. S1*A*) with the NCP, we observed the identical RAD51AP1-mediated mobility shifts by ethidium bromide first and then by Imperial protein stain (Fig. 1*B*, *lanes 4* and *5*). Of note, Imperial protein stain does not stain DNA (Fig. S1*B*, *lanes 1* and *2*). As determined by western blot analysis, RAD51AP1 enters the native gel in the presence but not in the absence of the NCP (Fig. 1*B*, compare *lanes 6* and *7* to *lane 8*), further supporting complex formation. We also expressed and purified His_6_-/FLAG-tagged human RAD51AP1 (Fig. S1*C*) and detected NCP binding activity for this protein as well (Fig. 1*C*, *lanes 3* and *4*, and *D*, *lanes 2* and *3*). Moreover, RAD51AP1 protein and both histone H2A and histone H3 were detected within the mobility-shifted bands (Fig. 1*C*, *lanes 5*–*10*, and *D, lanes 4*–*9*), suggestive of a tight RAD51AP1/NCP complex inclusive of octamer components. We also monitored NCP binding of purified human FLAG-tagged RAD54 (Fig. S1*D*), a member of the SWI2/SNF2 group of ATP-dependent chromatin-remodeling factors previously shown to efficiently bind to a core nucleosome with no DNA overhangs (15, 16). We found similar activities for both RAD51AP1 and RAD54 in NCP binding (Fig. 1*E*), further supporting the validity of our conducted experiments and obtained results. Purified human RAD51 (Fig. S1*E*), however, showed no affinity for the NCP, as determined by EMSA (Fig. 1*F*).

During DNA repair, nucleosomes are dynamically assembled and disassembled, guided by histone chaperones and ATP-dependent chromatin remodeling factors (17), and several DNA repair proteins with histone chaperone activity have been identified (18, 19, 20, 21). To test for the possibility that RAD51AP1 may function as a histone chaperone, we used a nucleosome reconstitution assay and purified human NAP1 (Fig. S1*F*), a protein with well-established histone chaperone activity (22, 23, 24), as a positive control. Our results show that, unlike NAP1, RAD51AP1 does not possess histone chaperone activity (Fig. S1*G*, *lanes 4–6*, and *H*, *lanes 4–8*).

### The C-terminal domain of RAD51AP1 is sufficient for NCP interaction

**Figure 2:**
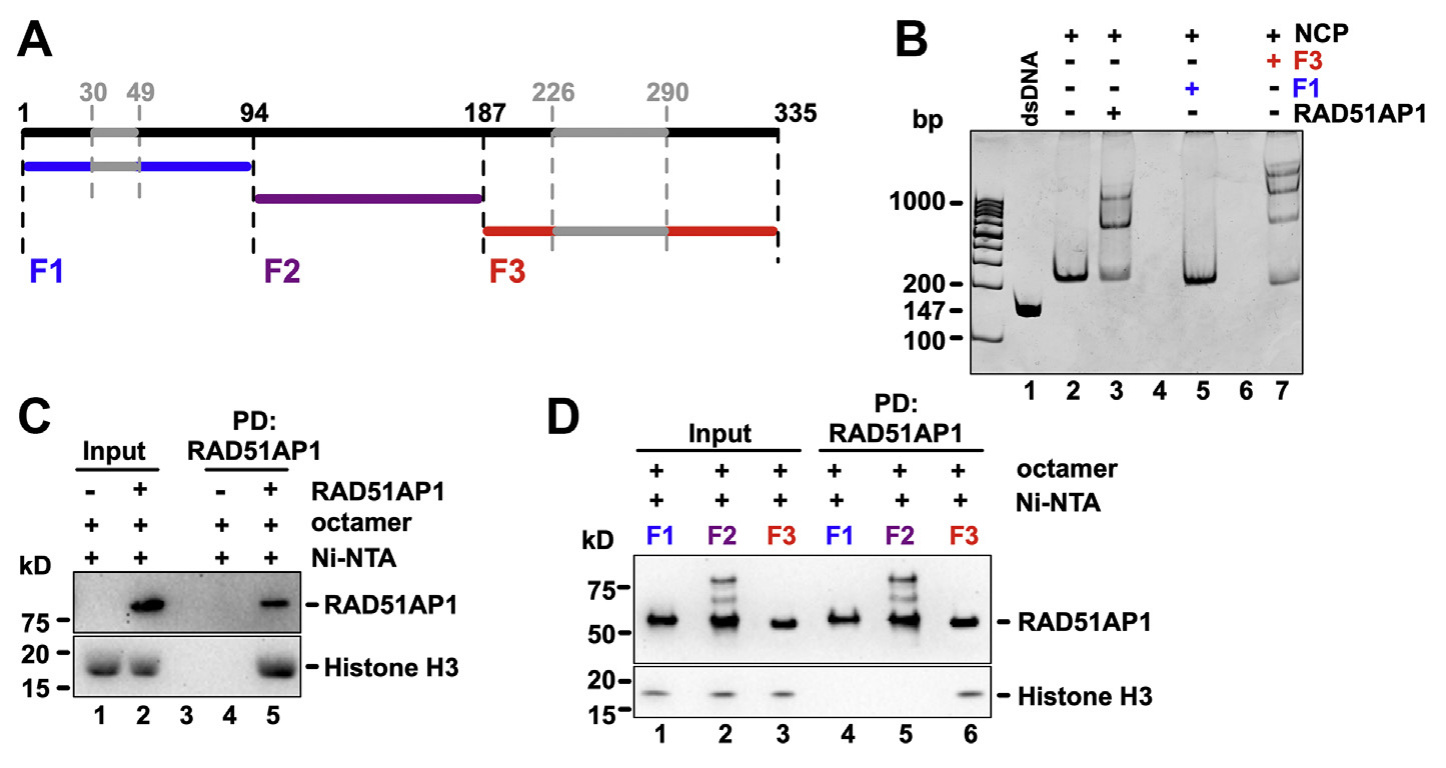
**The association between the NCP and RAD51AP1 occurs through the RAD51AP1-F3 domain.***A*, schematic of full-length RAD51AP1 (isoform two; in *black*) and RAD51AP1 fragments (F1 (*blue*), F2 (*purple*), and F3 (*red*)), as described earlier (7), including the two mapped DNA-binding domains (*gray*), as previously shown (4, 6). *B*, EMSA of MBP-/His_6_-tagged full-length RAD51AP1, -F1 and -F3 (0.2 μM each) with the NCP (0.2 μM) assessed by ethidium bromide. *C*, western blots obtained after Ni-NTA pull-down of MBP-RAD51AP1-His_6_ to show that RAD51AP1 *directly* interacts with histone H3 (lane 5). *D*, western blots obtained after Ni-NTA pull-down of the three MBP-/His_6_-tagged RAD51AP1 fragments to show that the interaction between RAD51AP1 and histone H3 occurs through the F3 domain (lane 6).

After confirming the novel association between RAD51AP1 and the NCP, we identified the domain in RAD51AP1 responsible for complex formation. We purified three previously described (6, 7, 8), nonoverlapping MBP-/His_6_-tagged fragments of the RAD51AP1 protein (F1, F2, and F3) (see Fig. 2*A* for schematic; Fig. S2*A*) and also full-length MBP-/His_6_-RAD51AP1 (Fig. S2*B*). RAD51AP1-F1 and -F3 each contain one mapped DNA-binding domain critical for protein function, as previously reported and identified using nucleosome-free DNA (6). We found that RAD51AP1-F3 is the only fragment capable of binding to the NCP (Fig. 2*B*, *lane 7*), whereas RAD51AP1-F1 and RAD51AP1-F2 are devoid of this activity (Fig. 2*B*, *lane 5*, and Fig. S2*C*, *lanes 5* and *6*, and *D, lanes 5* and *6*). Complex formation between RAD51AP1-F3 and the NCP was confirmed by gel filtration (Fig. S2, *E* and *F*). To determine the apparent binding affinities (*K*_D(app)_) of full-length RAD51AP1 to the 147 bp dsDNA fragment and the NCP, we performed EMSAs and quantified the free dsDNA or free NCP bands, respectively, after ethidium bromide staining (Fig. S2, *H* and *I*). After correcting for the diminished ability of ethidium bromide to intercalate into NCP-bound DNA (Fig. S2, *J* and *K*), we found the respective composite affinities of full-length RAD51AP1 to be exceptionally tight to dsDNA (*K*_D(app)_ = 12 nM) and the NCP (*K*_D(app)_ = 21 nM).

We assessed the possibility of a direct interaction between RAD51AP1 and the histone octamer (Fig. S2*G*) in affinity pull-down assays. We obtained evidence of a direct interaction between full-length RAD51AP1 and RAD51AP1-F3 with histone H3 (Fig. 2*C*, *lane 5* and *D*, *lane 6*, respectively). In contrast, neither RAD51AP1-F1 nor RAD51AP1-F2 interacts with histone H3 (Fig. 2*D*, *lanes 4* and *5*).

**Figure 3:**
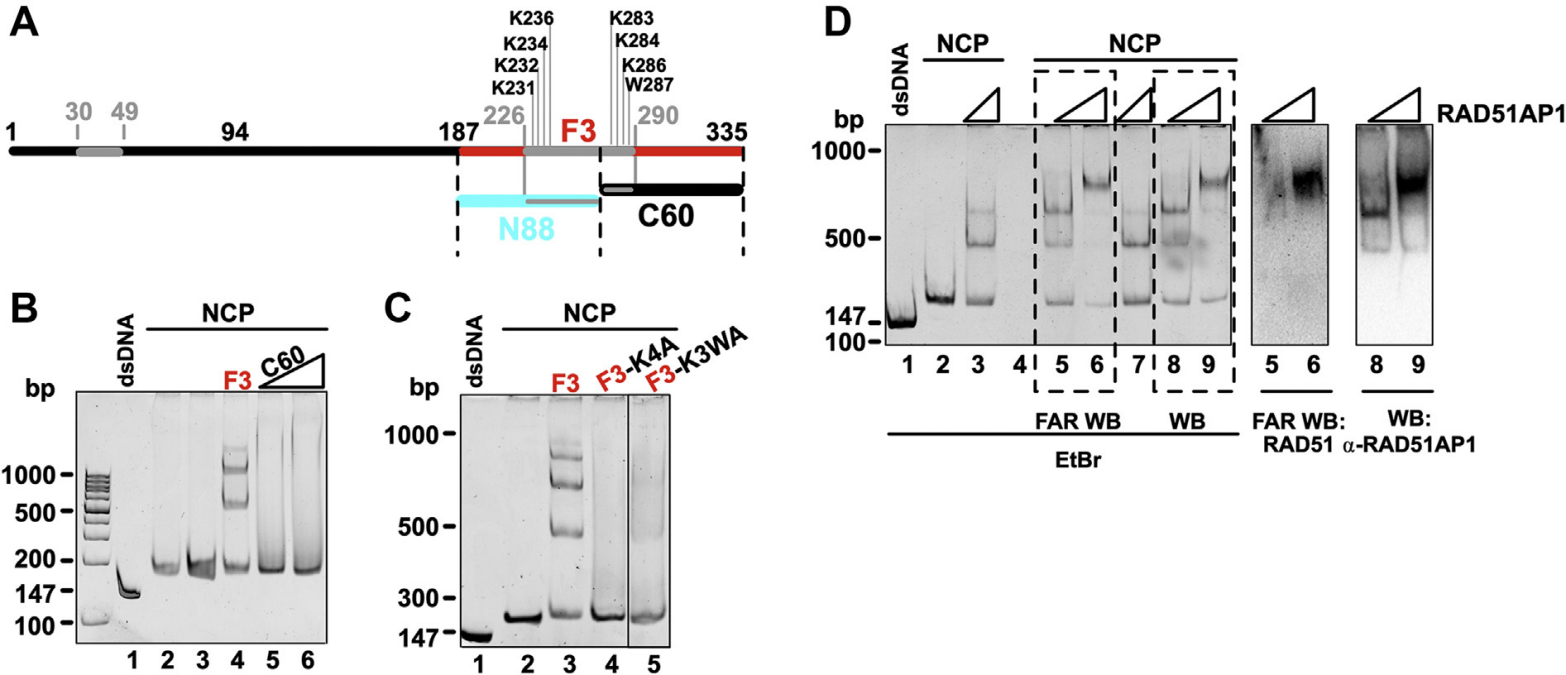
**The association between the NCP and RAD51AP1-F3 involves the DNA-binding domain.***A*, schematic of full-length RAD51AP1 (isoform 2; *black*), including the RAD51AP1-F3 (*red*), -N88 (*turquoise*), and -C60 (*black*) fragments with the mapped bipartite DNA-binding domain (*gray*) and its critical residues (∗K231, K232, K234, K236, K283, K284, K286, and W287), as previously identified (4, 6). *B*, EMSA of F3 (0.5 μM) and C60 (0.5 and 1 μM) with the NCP (0.4 μM). *C*, EMSA of F3 (0.5 μM) and DNA-binding defective mutants of F3 (F3-K4A and F3-K3WA; 1.0 μM each) with the NCP (0.4 μM). K4A: compound point mutant of K231A, K232A, K234A, and K236A; K3WA: compound point mutant of K283A, K284A, K286A, and W287A. ∗Residue numbering based on RAD51AP1 isoform 2. Lanes 1–5 are identical to lanes 1–4 and 7 in Figure S3*J*. *D*, western blots and FAR Western analysis after EMSA of full-length His_6_-/FLAG-tagged RAD51AP1 (0.25, 0.5 and 1.0 μM) with the NCP (0.4 μM) to visualize the presence of RAD51AP1 in shifted bands (lanes 8 and 9) and bound RAD51 to the RAD51AP1-NCP complex (lane 6).

Further division of the RAD51AP1-F3 fragment and its DNA-binding domain into an N-terminal 88 amino acid (aa) residues encompassing fragment (*i.e*., N88) and a C-terminal 60 aa residues containing fragment (*i.e*., C60) (see Fig. 3*A* for schematic; Fig. S3, *A* and *B*) impaired DNA binding, as expected ((6); Figure S3*C*, *lanes 4*–*7*). Moreover, neither C60 nor N88 was able to form a complex with the NCP (Fig. 3*B*, *lanes 5* and *6*, and S3*D*, *lanes 6*–*8*). Similarly, RAD51AP1-F3 in which several consecutive lysine residues and a tryptophan, previously and here deemed critical for binding to nucleosome-free DNA ((6); Figure S3, *E–H* and *I*, *lanes 5–7* and data not shown), were changed to alanine, lost or showed greatly reduced ability to bind to the NCP (Fig. 3*C*, *lanes 4* and *5*, and *S3J, lanes 4–9*). Collectively, these results show that the previously identified RAD51AP1 DNA-binding domain that is located in the F3 fragment (6) mediates complex formation between RAD51AP1 and the NCP and that contact is made by RAD51AP1 with histone H3 and, likely, nucleosomal DNA.

### The RAD51AP1-NCP complex retains the capability to bind to RAD51

Since our results confirmed that complex formation between RAD51AP1 and the NCP stems from the F3 region and since the F3 region contains the RAD51 interaction domain (4, 5, 25), we tested if full-length RAD51AP1 in the RAD51AP1/NCP complex was capable of retaining the interaction with the RAD51 recombinase. To this end, we conducted an FAR Western experiment in which we first separated the RAD51AP1/NCP complex by EMSA, transferred proteins to a PVDF membrane, and then incubated the membrane with purified human RAD51 protein before detection of membrane-bound RAD51 by western blot analysis. As described previously and shown in Figure 1, RAD51AP1 is present in shifted bands (Fig. 3*D*, *lanes 8 and 9*). In addition, RAD51 is bound and detected at the super-shifted RAD51AP1/NCP band (Fig. 3*D*, *lane 6*). As shown above, RAD51 by itself is unable to associate with the NCP (Fig. 1*F*). Collectively, these results suggest that the RAD51AP1 protein, while in complex with the NCP, retains the ability to interact with the RAD51 recombinase.

### RAD51AP1 promotes capture of the NCP in the duplex capture assay

**Figure 4:**
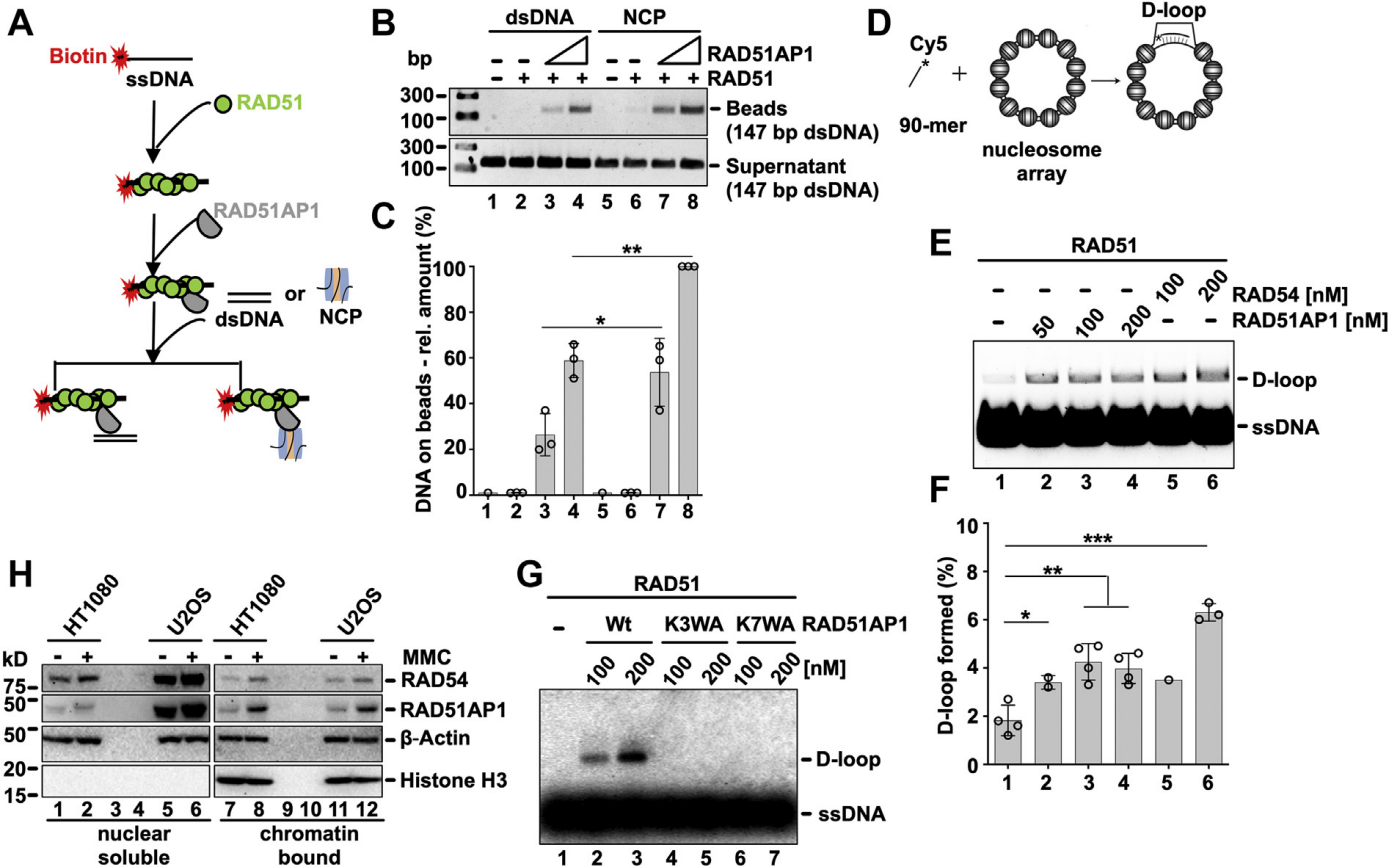
**RAD51 binds to the RAD51AP1/NCP complex, RAD51AP1 stimulates duplex capture with the NCP and is recruited to the chromatin fraction in human cells after DNA damage**. *A*, schematic of the duplex capture assay with His_6_/FLAG-tagged RAD51AP1 and either nucleosome-free DNA (*i.e*., 147 bp dsDNA) or the NCP. *B*, qualitative analysis of captured DNA (beads) and DNA in supernatant by agarose gel electrophoresis. *C*, quantitative analysis: *Symbols* are the results from independent experiments. Bars are the means from three independent experiments ±1 SD; ∗, *p* < 0.05; ∗∗, *p* < 0.01; two-way ANOVA. *D*, schematic of the D-loop reaction with chromatinized pBluescript II SK (-) plasmid DNA. *E*, addition of RAD51AP1 (50, 100 and 200 nM; lanes 2–4) or RAD54 (100 and 200 nM; lanes 5–6) promotes the RAD51-mediated D-loop reaction on chromatinized DNA. *F*, quantification of the results. Symbols are the results from independent experiments. Bars are the means from two to four independent experiments ± 1SD. ∗, *p* < 0.05; ∗∗, *p* < 0.01; ∗∗∗, *p* < 0.001; multiple *t* test analysis. *G*, agarose gel to show that wild-type RAD51AP1 (100 and 200 nM; lanes 2–3) promotes the RAD51-mediated D-loop reaction on chromatinized DNA, but RAD51AP1-K3WA (100 and 200 nM; lanes 4–5) and RAD51AP1-K7WA (100 and 200 nM; lanes 6–7) are unable to do so. *H*, western blots of fractionated extracts of the nuclei from HT1080 and U2OS cells without and after exposure to mitomycin C (MMC). The signals for ß-Actin and histone H3 serve as loading and fractionation control, respectively. RAD54 is shown for comparison purposes.

Earlier reports on RAD51AP1 showed its ability to stimulate duplex DNA capture in the context of nucleosome-free, naked dsDNA (8, 9, 26, 27). The results presented in this study show that RAD51AP1 interacts with the NCP and that the RAD51AP1/NCP complex retains the ability to interact with RAD51. These findings prompted us to test if RAD51AP1 would stimulate RAD51 activity in the duplex capture assay with an NCP. We tested the ability of RAD51AP1 to capture nucleosome-containing homologous template DNA *via* the NCP (see Fig. 4*A* for schematic of the assay). In accordance with our earlier findings (8, 9, 26, 27), addition of RAD51AP1 to the reaction stimulated the capture of nucleosome-free dsDNA (Fig. 4*B*, *lanes 3* and *4*). Interestingly, addition of RAD51AP1 also stimulated capture of the NCP (Fig. 4*B*, *lanes 7* and *8*). Quantitative analyses from three independent experiments show that RAD51AP1 prefers the NCP over nucleosome-free DNA in the duplex capture assay (Fig. 4*C, p <* 0.05 and *p* < 0.01; two-way ANOVA).

### RAD51AP1 promotes strand invasion of a chromatinized DNA template

Following duplex DNA capture, the RAD51 filament engages in synapsis and generates a displacement-loop (D-loop) by invasion of the homologous target sequence. RAD51-mediated strand invasion and the impact of accessory proteins on this activity can be measured *in vitro* by the oligonucleotide-based D-loop assay (28). In this assay, RAD51AP1 was previously shown to be a strong stimulator of heteroduplex DNA formation with nucleosome-free plasmid DNA (4, 5, 6, 9). Given RAD51AP1's affinity for the NCP, we were curious to test its stimulation of RAD51-mediated strand invasion on chromatinized template DNA (for schematic of the assay see Fig. 4*D*). We prepared the chromatinized pBluescript II SK(-) donor plasmid (Fig. S4*A*) and, in parallel to RAD51AP1, also assessed RAD54, a known stimulator of heteroduplex DNA joint formation with chromatinized target DNA (15, 29, 30, 31). Addition of 50–200 nM RAD51AP1 to this reaction stimulated D-loop formation approximately 2-fold (Fig. 4*E* and *F*, *lanes 2–4*; *p <* 0.05 and *p* < 0.01; multiple *t* test analysis). Addition of 100 and 200 nM RAD54 stimulated D-loop formation ∼2- and 3.5-fold, respectively (Fig. 4, *E* and *F*, *lanes 5–6*). We surmise that RAD51AP1 is able to stimulate RAD51-mediated joint-molecule formation with a chromatinized DNA template, although less efficiently than the RAD54 DNA motor protein.

Next, we tested if mutants of the F3 region in RAD51AP1 that abrogate the interaction with the NCP (Fig. 3*C*) would interfere with stimulating RAD51-mediated strand invasion into the chromatinized donor DNA. To this end we constructed two compound mutants in full-length RAD51AP1 (RAD51AP1-K3WA and RAD51AP1-K7WA; for schematic see Fig. S4*B*), purified these mutants (Fig. S4*C*) and tested their activity in the D-loop assay with chromatinized DNA. We found that both RAD51AP1-K3WA and -K7WA are completely defective in stimulating strand invasion into chromatin (Fig. 4*G*, *lanes 4–7*, and S4*E*, *lanes 5–7*, and *F, lanes 5–7*), although both mutants retained the ability to stimulate strand invasion into naked, nucleosome-free plasmid DNA (Fig. S4*H*, *lanes 4–7*). Together, these results provide strong evidence that the F3 domain in RAD51AP1 is critical for guiding homology search and joint-molecule formation within the context of chromatin.

### RAD51AP1 is recruited to the chromatin fraction in human cells after induced DNA damage

To assess the movement of nuclear soluble RAD51AP1 to the chromatin fraction in human cells, we fractionated nuclear extracts of HT1080 and U2OS cells that were grown under normal conditions or exposed to exogenous DNA damage by treatment with mitomycin C (MMC), an interstrand cross-linking agent that challenges HR DNA repair. Supporting our earlier findings obtained in HeLa cells (11), little RAD51AP1 is localized to the chromatin in HT1080 or U2OS cells cultured under normal growth conditions (Fig. 4*H*, *lanes 7* and *11*). However, upon exposure to MMC, increased amounts of RAD51AP1 protein are detected at the chromatin fraction in both HT1080 and U2OS cells (Fig. 4*H, lanes 8* and *12*). These results show that some activity of RAD51AP1 function is associated with the chromatin fraction in both HT1080 and U2OS cells.

## Discussion

HR involves the exchange of genetic information between homologous DNA molecules and is essential for preserving genome stability (1, 32, 33). The central steps of the HR reaction are the search for and the identification of a homologous DNA donor sequence and the formation of a joint molecule catalyzed by the RAD51 recombinase. Based on studies with nucleosome-free synthetic DNA substrates (4, 5, 6, 7, 8, 9), the RAD51 recombinase is assisted in this process by the accessory RAD51AP1 protein, for which a role in bridging or anchoring of the two molecules undergoing exchange has been discussed (4, 5, 9, 34). Yet, how exactly these steps are orchestrated in the chromatin environment in cells remains poorly understood.

**Figure 5:**
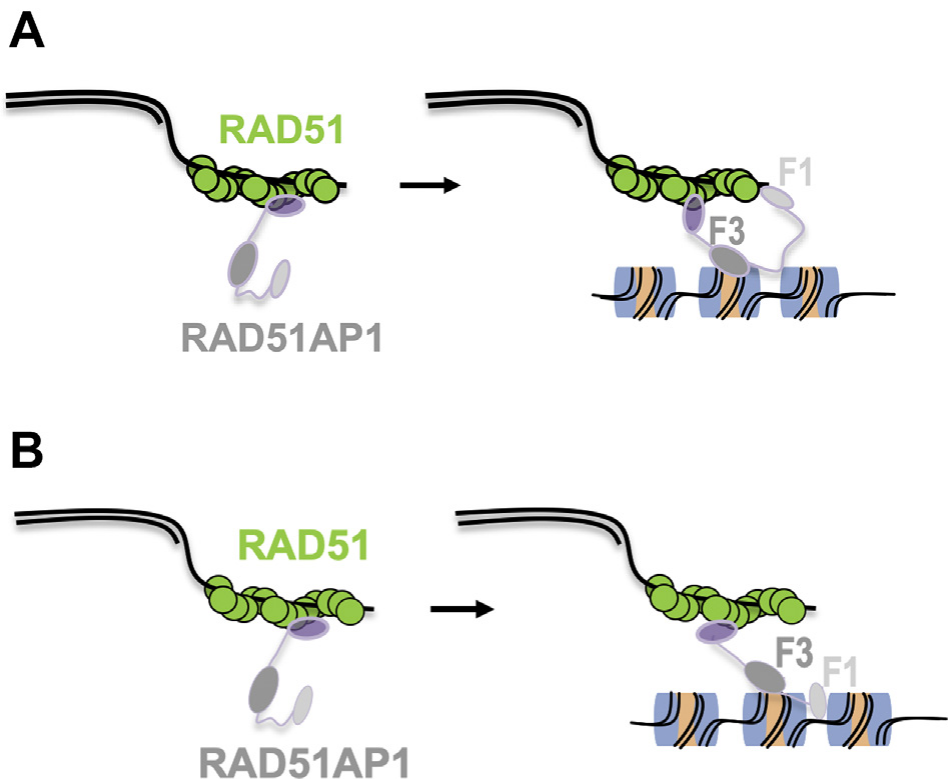
**Model depicting the role of the RAD51AP1-F1 and -F3 domains in ternary complex formation.***A*, to initiate ternary complex formation, F3 (*dark gray*) associates with a nucleosome on the incoming duplex DNA template, while F1 (*light gray*) binds to the ssDNA of the RAD51-ssDNA nucleoprotein filament. The very C-terminus of RAD51AP1 (*purple*) engages with RAD51, as previously shown (25). *B*, it is also possible that F1 binds to regions of nucleosome-free DNA within the dsDNA target.

Intrigued by our previous findings that showed binding of RAD51AP1 to a chromatinized DNA substrate *in vitro* (11), we were eager to test the affinity of RAD51AP1 to an NCP. To avoid any complications from direct binding of RAD51AP1 to overhanging free dsDNA, the NCPs that we used here were free of any linker DNA extensions. We show that RAD51AP1 avidly binds to the NCP and that complex formation is mediated *via* a C-terminal domain in RAD51AP1, a domain with previously identified DNA binding activity (4, 6). In contrast, RAD51AP1-F1, which contains the second and N-terminally located DNA-binding domain, has no ability in complex formation with the NCP. We also discovered a direct physical interaction between RAD51AP1-F3 and histone H3. Accordingly, we suggest that the RAD51AP1/NCP interface could be composed of RAD51AP1 residues in contact with the nucleosomal DNA and with histone H3, although other regions of contact cannot be excluded at this point. In our preferred interpretation of the results obtained, we suggest that the C-terminally located DNA-binding domain (in RAD51AP1-F3) associates with the nucleosomes of the homologous dsDNA target, while both the N-terminally located DNA-binding domain (in RAD51AP1-F1) and the peptide motif facilitating RAD51 interaction (25) associate with the presynaptic filament. As such, both DNA molecules may be tethered together to prepare for the exchange (Fig. 5). This model would be similar to the mechanisms of dsDNA capture proposed for Hop2-Mnd1 in meiotic recombination in yeast (35).

We detected at least two major shifted species when RAD51AP1 was incubated with the NCP. These observations suggest multiple contacts between RAD51AP1 and the nucleosome. It is possible that one species is mediated predominantly through contact with the nucleosomal DNA, while the second species associates predominantly through contacting histones. Further assessing the RAD51AP1:NCP stoichiometry in the future will be important to determine if two RAD51AP1 molecules can occupy one single nucleosome.

A select few proteins function synergistically with RAD51AP1 in biochemical HR assays based on synthetic nucleosome-free DNA substrates and also in cells. For example, RAD51AP1 interacts with UAF1 and the RAD51AP1-UAF1 complex works in conjunction with the RAD51 filament in duplex capture and synaptic complex assembly (26). As in RAD51AP1, a DNA-binding domain in UAF1 was identified that is critically important for HR-mediated chromosome damage repair (27, 36). Hence, it will be important to test if UAF1 shows affinity for nucleosome-containing DNA and if UAF1 can synergize with RAD51AP1 in the homologous pairing reaction with chromatin substrates. Similarly, functional synergism also was determined between RAD51AP1 and the tumor suppressor PALB2 (9). Notably, PALB2 does possess nucleosome-binding activity and contacts the nucleosome acidic patch (37, 38). Whether the nucleosome acidic patch also affects RAD51AP1 binding remains to be determined. Since most chromatin-binding proteins—to ensure the specificity of their activity—seem to recognize multiple chromatin interfaces (38, 39), we predict that the interaction between RAD51AP1 and chromatin is governed by multiple sites of contact and possibly other RAD51AP1-binding partners in cells.

## Experimental procedures

### Generation of the mononucleosome core particle (NCP)

NCPs were reconstituted using a 147 bp dsDNA fragment with positioning sequence (601 Widom fragment) and human histone octamers using salt gradient deposition (12, 14, 24).

### Electrophoretic mobility shift assay

The NCP or the 147 bp dsDNA (40) (0.2 μM each) and purified full-length RAD51AP1, RAD54, RAD51AP1-F1, -F2, -F3, -N88, -C60 fragments or RAD51AP1-F3 mutants (0.1–0.8 μM) were incubated in buffer A (50 mM Tris-HCl pH 7.5, 100 mM NaCl) at 4 °C for 30 min. Samples were fractionated by native 5% polyacrylamide gel electrophoresis (PAGE) in 0.2× TBE and 150 V for 1 h. Gels were stained by ethidium bromide (EtBr; Apex) or Imperial protein stain (Thermo Scientific) and image acquisition and analyses were performed on a ChemiDoc XRS+ instrument equipped with Image Lab 6.1 software (BioRAD).

### Purification of RAD51AP1, RAD51 fragments, RAD51AP1 mutants, and other proteins

The purification procedures for full-length human RAD51AP1, the RAD51AP1 fragments and mutants, and for human RAD54, RAD51 and NAP1 have been described elsewhere (5, 6, 11, 24, 41, 42). Purified human histone octamer was purchased from The Histone Source at Colorado State University (https://www.histonesource.com/) and purified as described (43).

For purification of the RAD51AP1-N88 protein, *E. coli* Rosetta cells harboring the plasmid were grown and induced for protein expression as described earlier (42). RAD51AP1-N88 was purified by tandem purification using Ni-NTA resin (Thermo Scientific) first, as described (42). Eluted protein was incubated with pre-equilibrated Strep-Tactin Superflow Plus (Qiagen) and gentle rotation at 4 °C for 1 h in binding buffer containing 50 mM Tris-HCl, pH 7.5, 150 mM NaCl, and 2 mM DTT. The resin was washed 4 × in three bed volumes of binding buffer, bound protein was eluted with binding buffer containing 2.5 mM d-Desthiobiotin (Millipore) and dialyzed into dialysis buffer (50 mM Tris-HCl, pH 7.5, 300 mM NaCl, 0.05% Triton X-100, 2 mM DTT and 20% glycerol) at 4 °C overnight before snap-freezing, and stored at –80 °C.

### Mutant construction

The generation of the RAD51AP1 fragments F1, F2, F3, and C60 is described elsewhere (6). K4A, K4A-1, K4A-2, K3WA, K3WA-1, K3WA-2 mutants of RAD51AP1-F3 and K4A and K7WA (*i.e.*, K4A with K3WA) mutants of the full-length RAD51AP1 protein were generated by Q5 Site-Directed Mutagenesis kit (New England Biolabs) following the instructions of the manufacturer and the primer pairs listed in Table 1.

**Table 1:** List of oligonucleotides/primers used in this study

Template/plasmid	Oligonucleotide	Sequence (5′-3′)
MBP-RAD51AP1-F3-His_6_ in pET24a	K4A forward	gcatccgcaTGTAATGCTTTGGTGACTTC^a^
	K4A reverse	agatgccgcCTCTTTCTTTTCTACTGGGG
His_6_-MBP-RAD51AP1 in pET24a	K4A forward	gcatccgcaTGTAATGCTTTGGTGACTTC
	K4A reverse	agatgccgcCTCTTTCTTTTCTACTGGGG
MBP-RAD51AP1-F3-His_6_ in pET24a	K4A-1 forward	AAAGAAAGAGgcggcaTCTAAATCCAAATGTAATGC
	K4A-1 reverse	TCTACTGGGGATTTTACC
MBP-RAD51AP1-F3-His_6_ in pET24a	K4A-2 forward	cgcaTGTAATGCTTTGGTGACTTC
	K4A-2 reverse	gatgcAGATTTCTTCTCTTTCTTTTCTAC
His_6_-MBP-RAD51AP1-K4A in pET24a	K3WA forward	tgcagcgGTCCCACCAGCGGCATCT
	K3WA reverse	ggtgccgcGCTTTCAGCTGAAGGACTGC
MBP-RAD51AP1-F3-His_6_ in pET24a	K3WA forward	tgcagcgGTCCCACCAGCGGCATCT
	K3WA reverse	ggtgccgcGCTTTCAGCTGAAGGACTGC
MBP-RAD51AP1-F3-His_6_ in pET24a	K3WA-1 forward	AGCTGAAAGCgcggcaCCTAAATGGGTCC
	K3WA-1 reverse	GAAGGACTGCGTATTTCTAATG
MBP-RAD51AP1-F3-His_6_ in pET24a	K3WA-2 forward	CAAGAAACCTgcagcgGTCCCACCAGC
	K3WA-2 reverse	CTTTCAGCTGAAGGACTG
pQE-TriSystem His_8_∙*Strep*2	N88 forward	TAAGCAGGATCCTGATTCTGAGGATGATTC
	N88 reverse	TAAGCAAAGCTTTATTTCTAATGGTTTCCTAGT
biotinylated-ssDNA	80-mer	TCGTAGACAGCTCTAGCACCGCTTAAACGCACGT AGGCGCTGTCCCCCGCGTTTTAACCGCCAAGGGG ATTACTCCCTAG
Cy5-ssDNA	90-mer	AAATCAATCTAAAGTATATATGAGTAAACTTGGT CTGACAGTTACCAATGCTTAATCAGTGAGGCACC TATCTCAGCGATCTGTCTATTT

a Mutated bases in lower case characters.

RAD51AP1-N88 was amplified from pOK24 (10) using the primer pairs listed in Table 1 and cloned from *Bam*HI to *Hind*III into pQE-TriSystem His∙*Strep*2 (Qiagen) to be expressed with an N-terminal *Strep*-tag and a C-terminal His_8_-tag.

### Western blot analysis

Western blot analyses followed our standard protocols (11, 44, 45, 46). The primary antibodies that were used are: α-RAD51AP1 (NB100-1129; Novus; 1:5000; and our own α-RAD51AP1 antibody, as previously described in (9)), α-RAD54 (F-11; sc-374598; Santa Cruz Biotechnology; 1:500); α-RAD51 (Ab-1; EMD Millipore; 1:4000), α-ß-Actin (ab6276; Abcam; 1:3000), α-H3 (ab1791; Abcam; 1:10,000), α-H2A (GTX1129418; GeneTex; 1:1000); α-FLAG (F3165; Sigma; 1:1000); α-MBP (PAI-989; ThermoScientific; 1:5000). HRP-conjugated goat anti-rabbit or goat anti-mouse IgG (Jackson ImmunoResearch Laboratories; 1:10,000) were used as secondary antibodies and SuperSignal Substrate kit (Thermo Scientific) for the detection of signal.

### FAR Western analysis

Following the EMSA, proteins were electro-transferred onto a PVDF membrane. The membrane was first soaked for 2 h in buffer I (10 mM KH_2_PO_4_, pH 7.4, 150 mM KCl, 15 mg/ml BSA, 2 mM 2-mercaptoethanol, 0.05% Tween-20) prior to incubation with 3 μg/ml purified RAD51 in buffer I and at 4 °C overnight. On the following day, the membrane was washed 3× in 10 ml buffer I, before incubation with primary α-RAD51 and secondary antibody (1 h each) in buffer I at room temperature and further detection of bound RAD51 protein by SuperSignal Substrate kit (Thermo Scientific).

### Histone chaperone assay

The histone chaperone assay was performed essentially as described (18). Briefly, 0.5 μM H2A/H2B and 0.5 μM H3/H4 were preincubated with increasing amounts of RAD51AP1 (1.4–22.4 μM) or NAP1 (1.2 and 5.0 μM) in buffer D (25 mM Tris-HCl, pH 7.5, 100 mM NaCl, 1.5 mM MgCl_2_, and 1 mM DTT) at 37 °C for 15 min. Then, the 147 bp DNA fragment containing the nucleosome positioning sequence (40) was added and samples were incubated further at 37 °C for 15 min. The reaction was stopped by the addition of 20% sucrose to a final concentration of 5%, and samples were separated on a native 5% PAGE in 0.2× TBE buffer for 1 h at 150V. DNA was visualized by EtBr staining and image acquisition was performed on a ChemiDoc XRS+ instrument equipped with Image Lab 6.1 software (BioRAD).

### Size exclusion chromatography (SEC)–micro-HPLC

The NCP (2.5 μM) and MBP-RAD51AP1-F3 (2.5 μM) were incubated in buffer E (50 mM Tris-HCl, pH 7.5, 100 mM NaCl) in a final volume of 50 μl at 4 °C for 15 min. Thirty microliters of this reaction was injected into a 2.4 ml prepacked analytical Superdex-200 Increase 3.2/300 gel filtration column (GE Healthcare) pre-equilibrated with buffer E. Gel filtration standard (1.35–670 kD, pI 4.5–6.9; BioRad) was used to ensure that the column was properly packed and the samples were evenly eluted. Chromatography was conducted at a flow rate of 0.075 ml/min and absorbance was monitored at 280 nm. Fractions of 0.1 ml were collected and analyzed by agarose gel electrophoresis and western blots for the presence of histone H3, DNA, and RAD51AP1-F3 to confirm coelution of the latter with the NCP.

### *In vitro* affinity pull-down assays

MBP- and His_6_-tagged full-length RAD51AP1 or MBP- and His_6_-tagged RAD51AP1-F1, -F2, -F3 (80 nM each) were incubated with pre-equilibrated Ni-NTA resin (Thermo Fisher Scientific) in buffer F (50 mM Tris-HCl, pH 7.5, 150 mM NaCl, 0.1% Triton X-100, 2% BSA) at 4 °C with gentle rotation for 1 h. The supernatant was removed and bound protein was further incubated with histone Octamer (80 nM) in buffer F containing DNase (1 U/μg protein) and at 4 °C with gentle rotation for 2 h. The resin then was washed 4 × with buffer F and bound protein complexes were eluted in 40 μl buffer F with 300 mM imidazole. Eluted complexes (10 μl) were fractionated on a 10% NuPAGE protein gel (Thermofisher Scientific), transferred onto a PVDF membrane, and detected by western blot analysis.

### Duplex capture assay

The duplex capture assay was performed essentially as previously described (47). Briefly, the presynaptic filament was formed by incubating 5 μl of streptavidin-coated magnetic resin (Roche Molecular Biochemicals) with 5′-biotinylated 80-mer ssDNA oligonucleotide (5 μM; Table 1) and RAD51 (700 nM) in buffer G (25 mM Hepes, pH 7.5, 50 mM Tris-HCl, pH 7.5, 35 mM NaCl, 45 mM KCl, 1 mM MgCl_2_, 0.16 mM EDTA, 2 mM ATP, 2% glycerol, 0.01% NP40, 0.4 mM ß-mercaptoethanol, and 100 μg/ml BSA) at 37 °C for 5 min. The resin was captured magnetically and washed once with 20 μl buffer G. The wash was removed and resin was resuspended in 10 μl buffer D containing full-length FLAG-tagged RAD51AP1 (400 or 800 nM). After a 5-min incubation at 37 °C, the resin was captured and washed as before. The supernatant was removed and 10 μl buffer G containing either the 147 bp dsDNA or the NCP (1 μM each) was added and incubated at 37 °C for 10 min. The resin was captured, the supernatant was saved, and the resin was washed 4× with 200 μl buffer G. Both resin and supernatant were treated with 2 mg/ml ProteinaseK in ProteinaseK buffer (2 mM Tris-HCl, pH 7.5, 1 mM CaCl_2_, 0.2% SDS) at 37 °C for 15 min, and supernatant-containing and resin-bound DNA were analyzed by 1% agarose gel electrophoresis. DNA was visualized by EtBr staining and image acquisition was performed on a ChemiDoc XRS+ instrument equipped with Image Lab 6.1 software (BioRAD). Quantification of signal intensities was done by ImageJ (https://imagej.nih.gov/).

### Chromatin assembly

Chromatin was assembled with human histone octamer by salt gradient dialysis on pBluescript II SK(-) plasmid DNA, as described (48). The plasmid/octamer ratio was based on 207 ± 4 bp DNA/nucleosome. The quality of the assembled chromatin was controlled by limited digestion with MNase.

### D-loop assay

The D-loop assay essentially was performed in 50 mM KCl and as described earlier (11, 42, 49), except that Cy5-labeled 90-mer ssDNA purchased from Integrated DNA Technologies (IDT) was used (Table 1). The 90-mer is homologous to the region of 1932–2022 bp within the pBluescript II SK(-) plasmid DNA (50). Fluorescence image capture and analysis were conducted on an Odyssey CLx Imaging System equipped with Image Studio 5.0 software (LI-COR).

### Cell culture, cell fractionation, and treatment with mitomycin C

U2OS and HT1080 cells were obtained from ATCC and maintained as recommended. Cell fractionation was carried out using the Subcellular Protein Fractionation Kit (ThermoFisher Scientific) and as described by the manufacturer. Exposure of cells to 1 μM mitomycin C (MMC; Sigma) occurred in regular growth medium at 37 °C for 24 h.

### Statistical analyses

Statistical analyses were performed using Prism 8 GraphPad Software on the data from two to four independent experiments. Statistical significance was assessed by two-way ANOVA or multiple *t* test analysis, as indicated. *p* ≤ 0.05 was considered significant.

## Data availability

All data are included in the article and in the supporting information. The raw data are available upon request.

## Supporting information

This article contains supporting information (6).

## Conflict of interest

The authors declare that they have no conflict of interest with the content of this article.
